# Effectiveness of a motivational intervention on overweight/obese patients in the primary healthcare: a cluster randomized trial

**DOI:** 10.1186/s12875-017-0644-y

**Published:** 2017-06-20

**Authors:** Juan Jose Rodriguez-Cristobal, Carlos Alonso-Villaverde, Jose Ma Panisello, Pere Travé-Mercade, Francisca Rodriguez-Cortés, Josep Ramon Marsal, Esther Peña

**Affiliations:** 1 Centre Atenció Primària Florida Sud. Direcció Atenció Primària Costa de Ponent-ICS. L’Hospitalet, Institut Universitari d’Investigació en Atenció Primària Jordi Gol (IDIAP Jordi Gol), Barcelona, Spain; 2 Cardiovascular Research Center, CSIC-ICCC, Hospital de la Santa Creu i Sant Pau (UAB), IIB-Sant Pau, Barcelona, Spain; 3 FUFOSA (Fundación Fomento Salud), Barcelona, Spain; 4 Univestitat de Barcelona, Facultad de Farmacia, Barcelona, Spain; 5 Unitat de Salut Mental Collblanc, L’Hospitalet del Llobregat, Barcelona, Spain; 6 Cardiovascular Department, Epidemiology Unit, University Hospital Vall d’Hebron. Lleida Research Support Unit, Primary Care Research Institut (IDIAP) Jordi Gol, Autonomus University of Barcelona, Barcelona, Catalonia Spain; 7 Cardiovascular Research Center, Hospital de la Santa Creu i Sant Pau, C/ Sant Antoni Ma Claret, 167 08025 Barcelona, Spain

**Keywords:** Overweight, Obesity, Motivational interview, Weight-loss, Cardiovascular risk factors

## Abstract

**Background:**

Overweight and obesity are common health problems which increase the risk of developing several serious health conditions. The main difficulty in the management of weight-loss lies in its maintenance, once it is achieved. The aim of this study was to investigate whether a motivational intervention, together with current clinical practice, was more efficient than a traditional intervention, in the treatment of overweight and obesity and whether this intervention reduces cardiovascular risk factors associated with overweight and obesity.

**Methods:**

Multi-centre cluster randomized trial with a 24-month follow-up included 864 overweight/obese patients randomly assigned. Motivational intervention group (400 patients), delivered by a nurse trained by an expert psychologist, in 32 sessions, 1 to 12 fortnightly, and 13 to 32, monthly, on top of their standard programmed diet and exercise. The control group (446 patients), received the usual follow-up.

**Results:**

Weight reduction was statistically significant in the second year with a mean reduction of 1.0 Kg in the control group and 2.5 Kg in the intervention group (*p* = 0. 02). While 18.1% of patients in the control group reduced their weight by more than 5%, this percentage rose to 26.9% in the intervention group, which is statistically significant (*p* = 0.04). Patients in the motivational intervention group had significantly greater improvements in triglycerides and APOB/APOA1ratio.

**Conclusions:**

The results highlight the importance of the group motivational interview in the treatment of overweight /obese patients in primary care, and in the improvement of their associated cardiovascular risks factors.

**Trial registration:**

ClinicalTrials.gov Identifier: NCT01006213 October 30, 2009.

**Electronic supplementary material:**

The online version of this article (doi:10.1186/s12875-017-0644-y) contains supplementary material, which is available to authorized users.

## Background

The prevalence of obesity is increasing steadily in developing countries and has become a serious public health issue. According to the World Health Organization (WHO), at least 500 million people worldwide were obese in 2008. They also estimated that by 2015, approximately 2.3 billion adults would be overweight and more than 700 million would be obese [1].For instance, according to the SEEDO 2011 study, the prevalence of obesity (Body mass index (BMI) > 30) in Spain is 14.5% (13.3% in men and 15.7% amongst women) while the overall overweight and obesity (BMI>25) is 53% [2–4]. Overweight and obesity in adulthood decrease life expectancy significantly and increase individual, national, and global healthcare costs [5, 6]. Obesity was estimated to account for between 0.7 and 2.8% [7] of a country’s total healthcare expenditures in several developed countries. When costs associated with being overweight (BMI > 25) were also included, the upper limit of this range increased to 9.1% [8] of total healthcare expenditures. The true costs are undoubtedly much greater as not all obesity-related conditions are included in the calculations. There is currently clear scientific evidence that obesity is a strong predictor of the risk of metabolic disorders such as dyslipidemia, hypertension, and diabetes, as well as a cause for potential premature mortality [9, 10]. Overweight and obesity are largely preventable. Favorable environments and communities are essential in determining people’s choices and in promoting healthier food choices and regular physical activity, and therefore preventing obesity [9, 11]. However, while significant weight loss can be achieved in a short-term, weight loss is notoriously difficult to sustain in the long term and it is for this reason that the research that would benefit these patients is of fundamental importance [4, 12]. It is crucial that patients integrate these life-habit changes into their lives. The Cochrane database study analyzed different studies aimed evaluate the effects of interactive computer-based interventions for weight loss or weight maintenance in overweight or obese people. The study concluded by saying that, compared to in-person interventions, interactive computer-based interventions result in smaller weight losses and lower levels of weight maintenance [13].

New guidelines released by the American College of Cardiology, American Heart Association Task Force on Practice Guidelines and The Obesity Society for the Management of overweight and obesity in adults that examined interventions to improve the maintenance of lost weight notify that, for all the trials that reported weight losses for two or more follow up evaluation periods, weight losses were consistently smaller in the long-term follow ups than at the 6- or 12-month assessments. These results indicate that further study is needed on facilitating maintenance of weight loss [14, 15].

Behavioural/ lifestyle interventions are effective at promoting initial weight loss [16–18]. However, successful in weight loss maintenance is often more difficult to achieve. Extended patient-therapist contact provides the opportunity to reinforce behavioral skills, support problem solving and providing continued accountability and motivation, and seems to be a key to diminish the weight regain [15].

The aim of this study (IMOAP: Group motivational intervention in overweight/obese patients in primary prevention of cardiovascular disease in the primary healthcare area) was primarily to assess whether a motivational group intervention (delivered by a nurse trained by an expert psychologist), was more effective than an isolated traditional intervention on weight loss and its maintenance, in overweight and obese patients. This was calculated as the percentage of patients reducing their weight by 5% and maintenance over time. And secondly, to determine whether this intervention was more effective in reducing cardiovascular risk factors associated with overweight and obesity.

## Methods

### Study design

This study design has been published elsewhere [19]. Briefly, this was a multi-centre cluster randomized trial of an intervention in overweight and obese patients, with a follow up of 24 months. Basic areas were randomly assigned to either control or intervention group as per a computer generated randomization schedule. The coordinators have contacted the Basic Health Areas (BHA), to explain the protocol and confirm their participation. Patients were recruited always as the five first who meet the inclusion criteria after centers were randomized. To avoid overburden the doctors, and nursing staff, patient recruitment and follow up were done in stages during the first six months of the study. The coordinators have contacted the BHA (24 clusters), to explain the protocol and confirm their participation. The distribution was: Intervention (12 clusters)/controls (12 clusters) with 20 patient of average size per cluster. The number of subjects necessary to divide into two independent groups has been calculated [19]. Basic Health Areas that dealt with the intervention groups received specific nursing-staff training from expert psychologists, consisting of a basic training strategy, and focusing on group motivation for life-style changes in overweight and obese patients. The training consisted of a number of workshops where role-play techniques were used to reinforce the concepts. The first patient was entered on September 2008. The final visit for the last randomized participant was planned for the last trimester 2010, with final study reports in the first trimester of 2013. Exclusion Criteria were: patients with severe clinical pathology (bedridden, dementia, advanced neoplasia, etc.), patients with secondary obesity (hypothyroidism, Cushing's disease, etc), patients with severe sensorial disorders capable of interfering with the motivational intervention, and patients with serious psychiatric disorders. They were included sequentially, from the beginning of the study. To avoid possible biases in the patients recruitment and follow up, and not overburden the nursing staff, the inclusion were developed in stages during the first six months of the study to the first two patients who met the inclusion requirements of the study and who present none of the exclusion criteria. This was carrying out superior quality control, using smaller sample size than would be possible if we randomized the patients.

### Screening and randomization

Patients included were aged between 30 and 70 years with overweight (BMI>25) or obese (BMI>30) of both genders, registered in the medical history (MH) or recently diagnosed.

The Jordi Gol i Gurina Foundation Ethics Committee in Barcelona approved the study and it was carried out in compliance with the Helsinki Declaration. Each IMOAP participant provided informed written consent using procedures reviewed and approved by the Ethics Committee review board.

### Control group

Four hundred and forty six participants followed the usual intervention, according to the protocols in each centre. Patients were visited every 3 months and doctors always included advice on life-style changes, physical exercise, hypo-caloric diet containing 1,200-1,500 kcal, and anthropometric measurements (weight, height and waist circumference). Follow-up blood tests by a healthcare professional were carried out at baseline, at 12-month and at 24-month follow-ups (triglycerides, APOA1, APOB-100, HDL cholesterol, and LDL cholesterol). In Additional file 1: Figure S1 is described the content of their visits.

### Motivational intervention group

Four hundred participants received the identical treatment as in the control group plus a group motivational intervention every 15 days, once fortnightly during weeks 1 to 12, and then monthly from week 13 to 32. Each session would last for one hour, for a 24-month follow-up period, with a total of 32 interventions, as described above. In Additional file 2: Figure S2 is described the contents of their visit.

### Outcomes and assessments

The primary outcome was the change in body weight at months 12 and 24 as compared with the control group. This was calculated as the percentage of patients reducing their weight by 5% and maintenance over time. Secondary outcomes included weight change in the intervention group as compared with control group and the percentage of participants whose initial weight was decreased by 5% or more at 12 and 24 months and by 10% or more at 12 and 24 months.

A wide range of interviews, physical examinations, and laboratory data were collected. The weight measurement (Kg) and waist measurement (cm) was always taken under the same conditions. Body Mass Index was calculated as: Weight in kilograms divided by height in meters squared (m^2^) (Kg/m^2^). The cardiovascular risk factors were also assessed: Hypertension was defined as either blood pressure readings of above 140/90 mm Hg on three occasions; Diabetes Mellitus: By case history or two prandial glycaemia readings > 126 mg/dl; -Smoking: n° packets/year.

### Statistical analysis

A complete cases analysis was performed. For quantitative variables, mean and standard deviation were used, whereas for the qualitative ones, proportions were used. The data analysis included an evaluation of the initial comparability of the patients receiving the two types of treatment using bi-variant techniques; the Chi-Square for the proportions and, in the case of the mean, the Student’s t-distribution or its nonparametric equivalent when necessary, by point estimation with a confidence interval of 95%. The effect of the treatment was estimated using a mixed lineal model. The clusters were defined by the primary centers whose gave care to the patients. A random-intercept model was adjusted. The intra-cluster correlation coefficient (ICC) also was calculated for the continuous bodyweight outcome.

## Results

### Baseline characteristics of the study participants

**Fig. 1:**
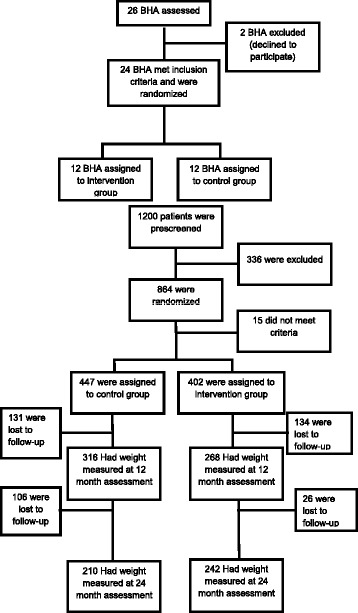
CONSORT flow diagram. Flow diagram of the progress through the phases of a parallel randomized trial of two groups

One thousand and two hundred patients were initially included. Trial Registration: ClinicalTrials.gov Identifier: NCT01006213. Study Start Date: January 2008. Eight hundred and forty six patients were randomized (77.19% women and 22.81% men). 52.72% formed the control group and 47.28% joined the intervention group. Mean age (±SD) was 55.49 ± 11.5 and 57.69 ± 22.1 years, with a mean body weight of 87.1±14.8 Kg, and 85.5 ± 13.9 Kg and a mean BMI of 34.1±4.8 and 34.1±4.8 (control and intervention group, respectively) (Table 1). There were no statistically significant differences in any demographic or lifestyle variables between the study groups at baseline. Approximately 60 and 47% of the participants in intervention and control group respectively had their weight measured at 24 months (Fig. 1).

**Table 1:** Baseline characteristics

	Usual Care (*n* = 446)	Motivational Intervention (*n* = 400)	All (*n* = 846)
	*mean (SD)*	*mean (SD)*	*mean (SD)*
Gender^a^			
Male	122 (27.4%)	71 (17.8%)	193 (22.8%)
Female	324 (72.6%)	329 (82.3%)	653 (77.2%)
Weight in Kg.			
At baseline	87.1 (14.8)	85.5 (13.9)	86.3 (14.4)
Height in cm.	159.8 (9.2)	158.4 (8.3)	159.1 (8.8)
BMI in Kg/m2	34.1 (4.8)	34.1 (4.8)	34.1 (4.8)
Waist circumference in cm.	107.7 (11.5)	107.6 (10.8)	107.7 (11.2)
SBP in mmHg	130.8 (14.8)	132.6 (15.2)	131.7 (15)
DBP in mmHg	78.7 (10.3)	79.3 (9.7)	79 (10)
Obesity^a^	374 (83.9%)	353 (88.3%)	727 (85.9%)
Diabetes^a^	76 (17%)	69 (17.3%)	145 (17.1%)
Hyperlipidemia^a^	269 (60.3%)	240 (60%)	509 (60.2%)
Smoking Habit^a^			
Never	298 (72.2%)	289 (77.3%)	587 (74.6%)
Current	54 (13.1%)	39 (10.4%)	93 (11.8%)
Ex-smoker	61 (14.8%)	46 (12.3%)	107 (13.6%)
Alcohol intake^a^	9 (2%)	3 (0.8%)	12 (1.4%)
Ischemic Heart Disease^a^	6 (3.2%)	1 (0.7%)	7 (2.2%)
Heart Failure^a^	6 (3.2%)	3 (2.2%)	9 (2.8%)
Transient Ischemic Attack^a^	1 (0.5%)	0 (0%)	1 (0.3%)
Neoplasm^a^	4 (2.1%)	3 (2.2%)	7 (2.2%)

^a^: n (%) and Chi-square Test, *SBP* Systolic Blood Pressure, *DBP* Diastolic Blood Pressure

### Weight loss

Body weight changes were evaluated at 12 and 24 months and are presented in Table 2. In the first year, participants in the control group lost a mean 1.3 Kg, those in the motivational intervention group lost a mean 1.8 Kg. The weight reductions were greater in the intervention group but did not reach statistical significance. In the second year, patients in the control group presented a loss of 1 Kg while the reduction in the Motivational Interventional group was 2.5 Kg this difference was statistically significant (*p*=0.02).

**Table 2:** Weight reduction between intervention groups

	Usual Care			Motivational Intervention				Fixed Model		Multi-level Model		
	N	Mean	SE	N	Mean	SE	OR	CI 95%	*p*	OR	CI 95%	*p*
Weight Baseline (kg)	428	87.1	0.7	393	85.5	0.7						
Weight. 1 Year (kg)	316	85.9	0.8	287	83.7	0.9						
*Weight difference (kg)*	302	1.3	0.1	283	1.8	0.4	0.45^a^	−0.47; 1.36	0.33	0.63^a^	−0.46; 1.73	0.26
*Weight ≤ Baseline* ^b^		179	59.3%		175	61.8%	1.11	0.80; 1.55	0.53	1.18	0.78; 1.83	0.44
*Weight Loss ≥ 5%* ^b^		50	16.6%		64	22.6%	1.47	0.98; 2.23	0.06	1.53	0.93; 2.56	0.09
*Weight Loss ≥ 10%* ^b^		12	4.0%		19	6.7%	1.74	0.84; 3.75	0.14	1.86	0.83; 4.80	0.15
Weight. 2 Years (kg)	210	84.9	0.9	242	83.2	1						
*Weight difference (kg)*	199	1.0	0.4	238	2.5	0.5	1.53^a^	0.31; 2.74	0.01	1.82^a^	0.32; 3.35	0.02
*Weight ≤ Baseline* ^b^		111	55.8%		156	65.5%	1.51	1.02; 2.22	0.04	1.62	0.98; 2.77	0.06
*Weight Loss ≥ 5%* ^b^		36	18.1%		64	26.9%	1.67	1.06; 2.66	0.03	1.74	1.03; 3.07	0.04
*Weight Loss ≥ 10%* ^b^		10	5.0%		19	8.0%	1.64	0.76; 3.75	0.22	1.67	0.67; 4.53	0.27

For each follow-up were calculated four variables:

*a) Weight difference* *was calculated as the weight in each visit minus the baseline weight, expressed in kg*

*b) Weight ≤ Baseline Weight* *: If the weight in the visit was lower than the baseline weight*

*c) Weight loss ≥ 5%:* *If the relative percentage loss of weight (weight difference/baseline weight) was ≥ 5%*

*d) Weight loss ≥ 10%* *: If the relative percentage loss of weight (weight difference/baseline weight) was ≥ 10%*

^a^differences of means

^b^n(%) and chi-square Test. *Kg* Kilograms, p: Mann-Whitney Test

**Fig. 2:**
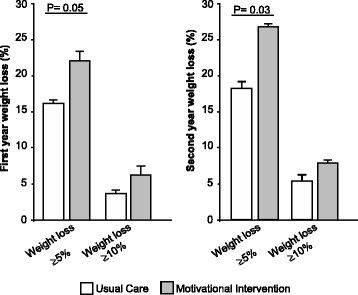
Effect of interventions on weight change. 5–10% of weight loss successful by group-*p* value for the comparison between

A 5% reduction of the weight in the motivational group intervention in the first year was one of the objectives set. We found that a higher number of patients reached this goal in the Motivational Interventional group than in the control group (22.6 versus 16.6 %) (Fig. 2). This difference was not statistically significant (*p*=0.09). In the second year the reduction in the control group was 18.1 versus 26.9 % in the Motivational Interventional group. This difference was statistically significant (*p*=0.04) (Fig. 2).

Weight loss of 10% was achieved by 4% in the control group and 6.7% of patients in the Motivational Interventional group (*p* = 0.1), which did not reach statistical significance after 1 year of intervention. Nor were losses significant in the second year (5 vs 8%; *p*=0.27) (Fig. 2).

The ICC was 0.08 for the bodyweight, so only the 8% of the variability was due by the center.

### Changes in cardiovascular risk factors

Participants who received the group motivational intervention had significantly greater improvements in triglycerides (*p*<0.0001) and apoB-100 (*p*<0.05) at the end of the second year, and in apoA1 (p<0.001; p<0.05) and apoB/apoA1 ratio (*p*<0.01; *p*<0.001) at the end of the first and also if the second year (Table 3).

**Table 3:** Changes in cardiovascular risk factors

	Usual care			Motivational intervention		
	Baseline^1^ mean ± SD ^*1vs4*^ *p-value=*	12 months^2^ mean ± SD ^*2vs5*^ *p-value=*	24 months^3^ mean ± SD ^*3vs6*^ *p-value=*	Baseline^4^ mean ± SD	12 months^5^ mean ± SD	24 months^6^ mean ± SD
Cholesterol	208.5 ± 39.8 *0.17*	206.4 ± 34.9 *0.78*	206.7 ± 33.6 *0.41*	211.5 ± 36.6	207.5 ± 36.4	203.8 ± 36
Triglycerides	135.4 ± (81.6) *0.2*1	134.7 ± 66.7 *0.08*	135.4 ± 65.6 *0.0001*	133.9 ± 93.9	127.6 ± 61.1	125.9 ± 65.1
HDL Cholesterol	54.6 ± 13.1 *0.66*	53.4 ± 13.5 *0.68*	54.2 ± 13.7 *0.41*	55.3 ± 13.4	53.8 ± 11.9	55.4 ± 14.3
LDL Cholesterol	131.6 ± 31.3 0.06	128.4 ± 33.8 *0.08*	126.4 ± 32.5 *0.06*	127.6 ± 32.6	125.9 ± 32.5	124.5 ± 35.9
APO A1	1.6 ± 0.3 *0.1*5	1.6 ± 0.4 *0.0002*	1.5 ± 0.3 *0.04*	1.6 ± 0.3	1.7 ± 0.3	1.8 ± 0.3
APO B-100	1 ± 0.2 *0.10*	1 ± 0.2 *0.30*	0.9 ± 0.2 *0.05*	1 ± 0.2	0.9 ± 0.2	0.8 ± 0.2
APOB-100/APO-A1	0.62 ± 0.2 *0.07*	0.62 ± 0.3 *0.002*	0.55 ± 0.3 0.0003	0.62 ± 0.3	0.52 ± 0.3	0.44 ± 0.3
SBP	130.8 ± 14.8 0.24	129.7 ± 15 *0.29*	131.7 ± 15.4 *0.57*	132.6 ± 15.2	130.9 ± 14.1	132.1 ± 14
DBP	78.7 ± 10.3 *0.44*	78.6 ± 9 0.12	77.0 ± 9.2 *0.17*	79.3 ± 9.7	78.2 ± 9.1	78.0 ± 8.8

*SBP* Systolic Blood Pressure, *DBP* Diastolic Blood Pressure. *P-value*: Mann-Whitney test

^1^UsualCare-Baseline

^2^Usual Care-12 months

^3^Usual Care-24 months

^4^Motivational intervention-Baseline

^5^Motivational Intervention-12 months

^6^Motivational Intervention-24 months

## Discussion

The main finding of this study was that in a motivational intervention group significantly more patients achieved a reduction of 5% or more of the initial weight, which is a common criterion for clinically meaningful weight loss [20–22]. Interventions that focus on changing eating habits and increasing daily physical activity in order to promote a healthy lifestyle are the best options to address overweight and obesity. In addition, other components of cognitive-conductive therapy from the behavioral perspective have shown to be relevant to be included in interventions targeting at overweight and obesity in order to improve their effectiveness and promote the maintenance of the obtained results. Moreover regarding the effectiveness of these interventions to reduce weight, evidence has found satisfactory results, achieving reductions between 5 and 10% of the initial weight [23–25].

This reduction was achieved in 22.6% of patients in the intervention group at the end of the first year but did not reach statistical significance. This loss was not only maintained in the second year but actually increased to 26.9% of patients and reached statically significance (*p*=0.04). Moreover, our findings suggest that the use of adequately motivational intervention for weight loss is also effective improving cardiovascular risks factors of obese/overweight patients. Furthermore, the significant increase in weight loss at year 2 in the intervention group could point out the efficacy of the group motivational approach among patients.

Weight loss in the intervention group was greater than the weight loss observed in other primary care studies [15, 19, 26, 27] but smaller than the weight loss observed by Wadden et al. in the first year of the study [20]. Nevertheless, an important difference was that, unlike the Wadden study, the loss recorded in our study was maintained in the second year. This group motivational intervention, added to usual care, has been shown to be a valid technique and should form a part of routine treatment in primary care in the future.

A variety of individual and group strategies have been used to enhance adherence in weight loss management. In a study conducted in 20 primary healthcare centers in Great Britain, investigators have demonstrated that weigh loss in 883 patients at high risk of cardiovascular disease through modifying fat intake, physical activity and smoking, had greatest benefit in the intervention group [28].

Shawk et al [29] performed a meta-analyses that include 36 clinical studies to assess the effects of psychological interventions that combined with dietary and exercise strategies, they concluded that combination of all strategies was more useful. Of all these thirty six studies only one was conducted in Spain in diabetic obese patients [30].

Smith-West et al [31] in a randomized, controlled study where all participants received an 18-month, group-based behavioral obesity treatment showed that obesity treatment was most beneficial when was adjunct to behavioral motivational interviewing for women with type 2 diabetes. Motivational interviewing was designed to help participants resolve ambivalent feelings that they may have about changing their behavior. Those in the traditional intervention group lost 3.1, 2.7, and 1.7 Kg at 6, 12, and 18 months, respectively. The addition of motivational interviewing increased weight loss significantly at each time point by 1.6 to 2.1 Kg.

The strengths of this study include the randomized design, and the provision of interventions by primary care therapist who treated overweight and obese patients in their ordinary local practices. This was a group intervention which permitted patient access to treatment, carried out by professionals in their own centers on patients whom they usually treat, without the need for external professionals. Patients received a hygiene-dietetic approach without pharmacological treatment. It is important to point out the high number of patients in the sample. Limitations included a considerable number of patients lost to follow-up. The higher drop-out rate (52.25 % completed the final visit at 2 years) observed in our study are in line with those reported in recent studies with a similar treatment duration. We did not specifically investigated the reason of drop-out and it is not possible perform a missing data analysis, but the majority of these losses were attributed to visit missing, patient deaths or changes in their address or in their general physician, which caused difficulties in their following-up. This is one of the major weak points in the follow-up of obese patients, similar to that found in other intervention trials, although far from the 86% of patients who completed the Wadden study [20]. It is notable that, in our study, adherence was better in the intervention group than in the control group with significantly fewer losses in the second year (33 vs 7%). This confirms the long-term efficacy of the group motivational approach among patients.

Modest weight loss—5 –10% of initial body weight has been shown to have a beneficial effect on cardiovascular risk factors associated with obesity and to improve risk factor clustering, such as improvements in blood pressure, and cholesterol [32]. Lifestyle intervention studies also suggest that modest weight loss can help to prevent or delay the appearance of type 2 diabetes and hypertension [33]. The beneficial effects of a moderate weight reduction apply not only in hypertension but also in the serum lipids levels. Vasankari et al [34] reported that the reduction of LDL-particles was proportional to the amount of weight lost. A weight loss of around 10% of the initial bodyweight was associated with a 33% reduction in the ratio of oxidized LDL to total LDL. A modest weight reduction has been shown to be beneficial in reducing the clustering of major cardiovascular risk factors. Individuals in the Framingham cohort, who lost at least 2.25 kg over 16 years, had a 40 –50% reduction in their total risk factor score. Recent retrospective analyses [35] from 401 patients conclude that patients losing only 5–10 % showed improvement on triglycerides, total cholesterol, and LDL cholesterol. This study supports the recommendation that 5–10 % weight loss may improve cardiovascular risk factors.

## Conclusion

Our study shows that combining usual care with group-based motivational interventions by a trained nurse, in addition to the regular follow-up appointments with a doctor currently offered to obese patients, significantly increases the maintenance of weight loss. Furthermore, also provides evidence that we may need to improve clinical practice and focus our efforts on psychological aspects that contribute to weight loss in obese and overweight patients. Even if this requires organizational changes, an effort should be made given that the benefits we have achieved justify this type of intervention.

## Additional files

Additional file 1: Content of Visits of the Control Group. (DOC 33 kb)

Additional file 2: Content of Visits of the Intervention Group. (DOC 30 kb)

## Abbreviations

BHA: Basic Health Areas
BMI: Body mass index
ICC: Intra-cluster correlation coefficient
IDIAP: Instituto de Investigación en Atención Primaria
IMOAP: Group motivational intervention in overweight/obese patients in primary prevention of cardiovascular disease in the primary healthcare area
WHO: World Health Organization
